# Exploring the Effects of Arts Intervention Groups on Well-Being Among Older Adults

**DOI:** 10.1093/geroni/igaa057.093

**Published:** 2020-12-16

**Authors:** Emily Ihara, Kathryn McNeil, Adriana Lopez-Piper, Maxine Eber, Catherine Tompkins, Holly Matto, Niyati Dhokai, Jatin Ambegaonkar

**Affiliations:** 1 George Mason University, Fairfax, Virginia, United States; 2 George Mason University, Vienna, Virginia, United States; 3 George Mason University, Manassas, Virginia, United States

## Abstract

Engaging in the arts reportedly improves well-being, but research is limited on the specific effects for community-dwelling older adults. The purpose of this randomized controlled trial was to examine how taking part in different arts interventions (dance & music), affects older adults’ overall well-being compared to a social conversation control group. Sixty-four participants (mean = 71 years old) participated twice weekly in a 10-week intervention that included ballroom dancing (n=23), ukulele playing (n=17), and social conversation (n=24). At the conclusion, three focus groups were held to assess participants’ experiences and subjective evaluation of the interventions’ impact. Twenty-two out of the sixty-four participants (dance= 8, ukulele = 6, social conversation = 8) took part in focus groups. Transcripts of the recorded focus groups were independently coded and compared. Common themes were agreed-upon by two researchers. Focus groups revealed positive outcomes for participants in all three groups. Several themes emerged across the intervention groups compared to the control group, including participants feeling challenged as they crossed their comfort zones, reporting increased confidence, enhanced social connections, and a sense of accomplishment when learning new skills. Community-dwelling older adults reported improved health-related outcomes after taking part in arts and social conversation sessions. Implementation of community-engaged arts intervention programs for older adults in the future may examine motivators which attract participants, foster positive social connections during sessions, and use participant-empowering pedagogical adaptations to retain participants. These factors can increase the efficacy of arts-engaged programs and help improve well-being in older adults.

